# The influence of community factors in the implementation of community-based interventions to improve antenatal care: a qualitative study based on the IMCHA programme in Tanzania

**DOI:** 10.1186/s12978-021-01225-5

**Published:** 2021-09-22

**Authors:** Chakupewa Joseph, Stephen O. Maluka

**Affiliations:** 1 Department of Development Studies, History and Political Science, Mkwawa University College of Education (MUCE), P.O. Box 2513, Iringa, Tanzania; 2 Institute of Development Studies, University of Dar es Salaam, P.O. Box 35169, Dar es Salaam, Tanzania; 3 Dar es Salaam University College of Education (DUCE), Dar es Salaam, Tanzania

**Keywords:** Antenatal care, Community-based interventions, Context, Tanzania

## Abstract

**Background:**

Efforts to improve antenatal care have been heightened to reduce global maternal deaths. In resource-limited settings, community-based interventions play a pivotal role in improving antenatal care services. However, effective implementation of community-based interventions is influenced by prevailing community-related factors. Drawing from the community-based interventions implemented in Iringa Region in Tanzania, this paper underscores how community factors influence implementation and ultimate improvement of antenatal care services.

**Methods:**

A qualitative case study design was employed using in-depth interviews, focus group discussions and document reviews. Data was collected in Kilolo and Mufindi districts in Iringa Region where community-based interventions were implemented. A total of one hundred and forty-six (146) participants were involved in the study. Eighty-six (86) participants were interviewed and sixty (n = 60) participated in focus group discussions. Data were analysed thematically and manually by categorizing and coding emerging issues to facilitate analysis and interpretation.

**Results:**

Key factors that influenced the implementation of the community-based interventions were the community readiness to adopt the interventions and effective local administrative systems. Stakeholders’ engagement and local health system support were also pivotal for improving antenatal care services. However, the physical environment, bullying of implementers of interventions and family-related challenges constrained the implementation of the interventions.

**Conclusion:**

This study has shown that the performance of community-based interventions is highly influenced by community-related factors. More specifically, inadequate community engagement may lead to community members’ reluctance to adopt implemented interventions. Therefore, in-depth understanding and adequate management of community engagement are important during the planning, development and implementation of community-based interventions.

## Plain English Summary

Increasing uptake of antenatal care services is crucial for improving maternal and child health. One of the strategies that has been found to be relevant for improving uptake of antenatal care services in resource constrained countries especially in rural communities is implementation of community based initiatives. This study focused on the project that was implemented in Iringa region, Tanzania under the Innovating for Maternal and Child Health in Africa (IMCHA) program, which was implemented through community based initiatives.

In order to understand community contextual factors that facilitated or constrained implementation of the IMCHA project, a participatory approach was applied to seek views from women groups as key project implementers and other stakeholders such as community leaders, male champions’ religious leaders, health care providers and health facility governing committees. A total of one hundred forty six people were engaged in interviews and focused group discussions.

Community readiness to adopt the interventions emerged as a determining factor for success of the project. Stakeholder engagement also enabled participatory implementation. The major constraining factors were the physical environment that made navigation through the villages difficult as well as negative feedback from family members.

This study concludes that community readiness and participatory approaches can be enhanced by various aspects integrated in the initial designing of project activities. This strengthens the project support system critical for sustainability.

## Introduction

Efforts to improve maternal and child health have been long prioritised at the local and global level. This is precipitated by the fact that preventable deaths caused by pregnancy-related complications are still recorded globally. Evidence shows that everyday 830 women die worldwide from pregnancy and birth-related complications, with 99% of the deaths occurring in low and middle-income countries (LMICs) [1]. The average maternal mortality ratio (MMR) in Sub Saharan Africa (SSA) stands at 510 per 100,000 live births, which raises doubts as to whether the targeted goal of reaching less than 70 per 100,000 live births will be achieved by 2030 as echoed in the Sustainable Development Goals (SDGs) No. 3.1 [2]. In Tanzania, for instance, between 2015 and 2016, maternal mortality was 556 per 100,000 live births; which was higher compared to 454 per 100,000 live births in 2010 [3]. Effective adherence to Antenatal Care (ANC) can potentially reduce adverse effects during pregnancy and after delivery. In particular, ANC provides a platform for critical healthcare function, health promotion and prevention thereby enabling screening and appropriate diagnosis of risks that may affect pregnant mothers [2, 4]. Additionally, pregnant women who attend ANC as recommended have the potential to avert any complications as well as access to preventive interventions like tetanus toxoid immunization, intermittent preventive treatment of malaria, deworming, iron and folic acid; among other benefits [5].

The World Health Organisation (WHO) recommends that pregnant women without health complications should attend at least eight ANC visits with the first attendance being within the first trimester [6]. This is seen as improvement of the former ANC guidelines that recommended at least four ANC visits in the absence of complications [7]. Some LMICs including Tanzania still follow four or more ANC visits for pregnant women not diagnosed with severe complications [8]. Despite various efforts in most LMICs, attendance of ANC is still below the desired levels. In Tanzania, for instance, only 24% of pregnant women attended the first ANC visit within the first trimester, whereas 51% of them attended four times or more [2, 9]. This trend is prevalent in other LMICs; for instance, in Ethiopia, only 18% of pregnant women attended ANC early in 2014, with 32% of them attending four times as recommended [10], while in Uganda, only 21% of pregnant women attended ANC early, with 48% of them attending four times or more [11]. Given the status in various countries, ensuring attendance of ANC is still critical.

In order to address the causes leading to delays in attending ANC and completion of recommended visits, different strategies have been implemented in LMICs, including Community-Based Interventions (CBIs). CBIs refer to sets of interventions designed to influence changes in community infrastructure, services, norms, attitudes, beliefs and policies that would result in improved health status of community members [12]. In the course of improving ANC and overall maternal and child health (MCH) services, CBIs are commonly implemented through home visits and women participatory learning and action groups [13]. Empirical evidence shows that CBIs have the potential to improve ANC and Maternal and Child Health (MCH) services in poor resource settings [14–16]. For instance, in Nepal, CBIs lowered neonatal mortality by 30% and maternal mortality by 80% per 100,000 live births [17]. Similarly, Maikhanda interventions in Malawi reduced neonatal mortality ratio by 22% and perinatal mortality by 16% [18] whereas interventions implemented in Jhankand and Orrisa in India between 2009 and 2012 lowered maternal deaths by 32% and maternal depression by 57% [19]. However, not all community-based interventions successfully manage to achieve targeted goals. In Bangladesh, for example, CBIs did not lead to significant reduction in maternal and child health, and stillbirths did not differ between intervention and control clusters [20].

Contextual factors have a determining role in facilitating and/or constraining implementation of community-based interventions [21–24]. In fact, the context in which interventions are implemented has an impact on the potential user of the interventions, implementers of the interventions, and determines both professional and organizational outcomes [25–27]. Scholars have underscored different types of contexts that may influence implementation of CBIs. These are categorised into (1) external context, which includes policy and legislations and buy-in by internal and external stakeholders (2) organisational context, which includes organisational culture, leadership and resources (3) professional context, which subsumes roles and competency and (4) interventions like nature, characteristics and complexity [28]. Community Contextual Factors (CCF) are also recognised as a strategy for maximizing performance of WGs interventions to improve ANC services [28, 29]. This is because community-based interventions are embedded within the community systems that affect the ability of interventions to realize community changes.Community systems also help implementers of interventions, implementation research team and other stakeholders to anticipate barriers and problems before they arise [29]. A study by Kok and colleagues identified several CCFs that influence the performance of interventions including socio-economic factors, cultural norms, values, practices and beliefs; gender roles, disease-related stigma, safety and security as well as education and knowledge level of the targeted groups [30]. Other CCFs which have also been reported as constraining the performance of interventions include effective use of the existing community resources especially social norms and values [31], long-distance to health facilities and lack of affordable and accessible transport [32].

While community factors are important in the implementation of community-based interventions, there is scant literature on the subject from LMICs. For instance, most of the existing literature addresses holistic contextual factors covering all aspects of context thereby limiting in-depth analysis [14, 23]. Moreover, literature that addresses CBIs in Tanzania is insufficient with few studies enlisted [33, 34] while literature on CCFs is virtually missing. This study, therefore, explored the influence of community-related factors on the performance of community-based interventions through women participatory groups in Iringa Region.

## Methodology

### Study design and setting

This study employed a qualitative case study design because it aimed at examining the implementation of CBIs in the real-life context of rural intervention villages while reflecting upon the perspectives of the participants [35, 36]. The study was conducted in Kilolo and Mufindi districts in Iringa Region which were implementing a large project under the Innovating for Maternal and Child Health in Africa (IMCHA) programme (2015–2020). The IMCHA project sought to improve maternal and child health by increasing community demand for ANC services while improving health service delivery at the facility level. The two districts were selected because they exhibited unacceptably low ANC uptake. In 2018, for instance, the first ANC attendance records within twelve weeks in Kilolo and Mufindi districts were 16.8% and 27% respectively. In the same year, pregnant women who completed four or more ANC visits constituted 27.1% in Kilolo District and 23% in Mufindi District. Table 1 shows the key characteristics of the study settings which may account for the situation.

**Table 1:** Key characteristics of the study settings

	Kilolo district	Mufindi district
Population	218,130	265,896
Division	03	05
Wards	22	28
Villages	106	121
Public hospitals	0	0
Health Centre	2	8
Dispensaries	56	45
Health workers available	61%	62%
Shortage of health workers	39%	38%
ANC Attendance within 1st trimester as of 2016	16.8%	27%
Completion of four or more ANC visits as of 2016	27.1%	23%

Source: CCHP Mufindi, 2018 and CCHP Kilolo, 2018

### Participatory action research under IMCHA

**Fig. 1:**
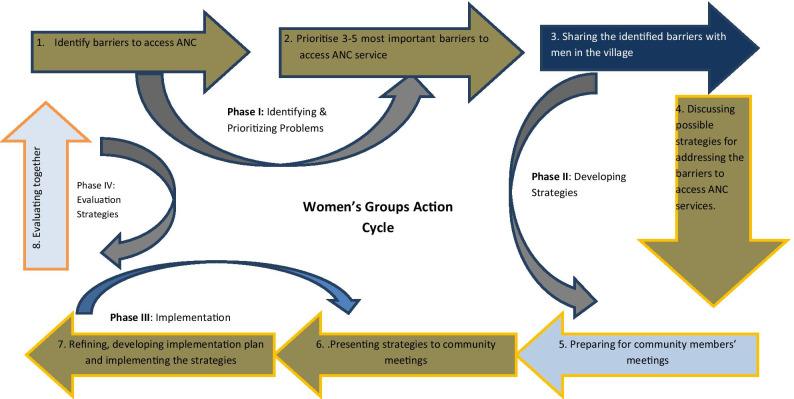
Women groups participatory action cycle

The IMCHA project was implemented through Participatory Action Research (PAR) in which the Implementation Research Team (IRT) worked together with community members to address MCH challenges with emphasis on ANC. The PAR was implemented through a series of meetings with implementers of the interventions, namely Women Groups (WGs), Male Champions (MCs), Women Groups Supervisors (WGS) and other stakeholders. The PAR was facilitated by the IRT from the University of Dar es Salaam and health managers from Iringa Region. The phases and series of meetings are indicated in Fig. 1.

As shown in Fig. 1, Phase I covered identification of ANC problems facing respective villages and prioritised the most 3–5 ANC problems affecting the community. Phase II involved developing strategies to address the prioritized ANC problems. The proposed strategies were presented to the community meetings by WGs involving different stakeholders, namely government and community leaders, religious leaders, health care providers and health facility governing committees. In phase III, WGs implemented the proposed strategies to improve ANC services. The dominant strategies that were developed included conducting community sensitisation meetings, visiting households with pregnant women, male champion engagement, engaging community gatekeepers such as religious leaders and village elders as well as health care providers (HCPs). In Phase IV, the implementers of interventions evaluated the effectiveness and sustainability of the implemented strategies. Details of the PAR process have been reported elsewhere [37].

### Study site selection and recruitment of participants

The study was conducted in selected wards in Kilolo and Mufindi districts. In each district, five wards were selected; In Kilolo District, the selected wards were Lugalo, Ibumu, Ng’uruhe, Mlafu and Ukumbi. In Mufindi District, the selected wards were Kibengu, NyololoShule, Itandula, Igowole and Kasanga. In each ward, two villages were selected to implement the interventions. Participants in this study were purposively sampled from those who directly participated during the implementation of the interventions. Interviews and FGDs were conducted until saturation point was reached meaning that no new information was emerging in the successive interviews and FGDs. As indicated in Table 2, a total of 86 participants were involved in the study.

**Table 2:** Study participants

S/N	Category of participants	Number per district	
		Kilolo	Mufindi
1	Women groups members	15	15
2	Male champions	05	05
3	Women group supervisors	05	05
4	Community Gatekeepers	03	03
5	Health care providers	04	04
6	Village management team	05	05
7	Council health management teams	04	04
8	Implementation research team	04	
	Total	86	

Source: Field Data (2019)

### Data collection techniques

The first author (CJ) collected data using in-depth interviews (IDIs), Focus Group Discussions (FGDs) and document reviews. The interviews enabled collection of detailed information regarding CCFs and provided insights on how various factors influenced the implementation of WGs interventions. Interview guides were developed after reviewing the IMCHA technical reports and grant proposal. Interview guides were developed to suit each category of participants. The required information pertained to the community readiness to adopt the designed strategies, the role of local leadership, stakeholders’ engagement and the role of the local health system. Interviews were conducted from June to September 2019 and each session lasted between 30 and 45 min. The interviews were tape-recorded with permission from the participants.

Focus group discussions were conducted with WGs members and Women Group Supervisors who were actively involved in the implementation of the community-based interventions. In total, 60 people participated in six (6) FGDs. Three (3) FGDs were conducted in each district with 10 participants each. On average, discussions lasted between 45 min and 1 h. The discussions enabled the participants to share their experiences during the implementation of the WGs interventions. The data collected from FGDs revolved around the community factors that facilitated or slowed down the implementation of WGs interventions.

In addition, evidence from documents was collected in order to gain an understanding of health status as well as community context factors influencing the implementation of the interventions. A document review guide was used and documents were selected based on the pre-identified themes, which included global and national information on ANC and CBIs, and country-specific reports from relevant departments and sectors. The reviewed documents included Comprehensive Council Health Plans (CCHPs), which provided information on the status of maternal and child health, antenatal care and overall health services provided in the two study districts. Other documents included IMCHA project strategic and action plans, programme meeting reports, formative research reports, workshop reports and project progress reports. These reports provided a good opportunity for detailed analysis on how WGs interventions were implemented, recorded successes, the challenges experienced as well as the perceptions of the community members on the interventions. On the whole, the documents enabled comparison, triangulation and confirmation of the findings generated during in-depth interviews and FGDs.

### Data analysis

Thematic approach was used to analyse the data [38]. Data from in-depth interviews and FGDs were transcribed verbatim and translated from Swahili into English. Transcripts and field notes were read several times in order to identify common patterns which were then crosschecked with tape-recorded information. All transcripts were discussed among the research team members to ensure appropriate interpretation of the received information. Common ideas that emerged included community readiness, the role of local administrative systems, health system factors, community physical environment, bullying of WGs by the community members and WGs family-related challenges, among others. These common ideas were coded and categorised into common themes, namely community facilitators and barriers of the WGs interventions.

### Ethical considerations

The study was approved by the ethics committees of the University of Dar es Salaam in Tanzania with certificate No AB3/14 (B). Permission was sought from the Iringa Regional Administrative Secretary and the respective offices in Kilolo and Mufindi districts. Verbal informed consent was obtained from participants because in these rural settings asking respondents to sign consent forms would be quite intimidating. In addition, some respondents were illiterate and could not sign written consent forms. Participants were informed of their right to withdraw from the interviews for any reasons at any time. Interviews were audio-recorded after obtaining consent from the participants. Individual identification was not attached to the findings and all quotes used to illustrate participants’ views did not have any personal identifiers.

### Findings

The findings reported in this paper are broadly categorised into two major themes, namely facilitators and barriers to the implementation of the WGs interventions. On one hand, facilitating factors include community readiness to adopt the interventions, the role of community leaders, stakeholders’ engagement and support from local health systems. On the other hand, constraining factors include the unfavourable physical environment, victimisation of the WGs and family-related challenges.

## Community-related factors that facilitated the implementation of WGs interventions

### Community readiness to adopt the interventions and facilitating aspects

During the implementation of the community-based interventions, community members showed satisfactory readiness to adopt the strategies implemented by the WGs. Analysis of data showed a high level of community ownership through active participation in the interventions, knowledge and adoption of the implemented interventions. For instance, the community readiness to adopt the interventions was seen in the manner in which the community elected women groups and women group supervisors who they considered fit to implement the interventions. One of the women group supervisors had this to say:

*Members in this community were anxious for this intervention, when they were called upon to select women group supervisors, they participated actively and sometimes identified even absent members who they thought would be good performers. They were really eager to have the interventions rolling (IDI with WGS in KDC).’*

The above statement reveals that the community was ready to adopt the intervention and actively participated in activities that would ensure this process. Additionally, community leaders revealed that community members were always willing to participate in community activities when mobilised. They came for the educative and entertaining messages that women groups were passing on. The community readiness to adopt interventions enabled the women groups to implement the expected activities in a timely manner; and this was facilitated by a number of aspects. For instance, when asked to mention the factors for community readiness, participants mentioned active participation during sensitisation meetings as one of  the key factors that triggered their readiness. It was revealed that during these meetings, community members were sensitised on various issues including, among others, the importance of attending ANC within the first trimester, completing four or more ANC visits, using family planning methods and avoiding home delivery. During sensitisation meetings, community members had the opportunity to ask questions, seek clarification on specific concerns and probe more about ANC from WG members. This is illustrated by one respondent:

*Whenever we conducted sensitisation meetings, community members were always willing to attend. In fact, there were few instances when attendance was poor; but this was due to other factors, especially unfavourable weather. In these meetings, participants were active and raised so many maternal-related issues. They wished that these interventions could have taken place many years back* (IDI with WGs members in KDC).

Another factor for community readiness was the direct support provided by the implementers of the interventions to pregnant women who needed help. This increased community confidence and trust in the implementers of the interventions; and this simplified community adoption of the implemented interventions. It was revealed that the implementers of the interventions mostly WGs provided material support such as sugar, cooking oil, soap and flour, in addition to accompanying pregnant women who needed facility assistance. During FGDs, participants explained how this touched community members as narrated by one participant.

*At first, community members were skeptical about our work; they thought we were not serious enough. But having witnessed our support for  pregnant women and our search for malnourished children in the villages, their confidence in us increased. Afterwards, it was the community members themselves who used to direct us to pregnant women whom they thought needed our assistance* (FGD with WG, in KDC).

Participants also revealed that following the implementation of the interventions, pregnant women were keen to complete four or more ANC visits, and even their spouses were supportive. It was evident that some community members who were skeptical about using antenatal care services started using them. For example, men could also accompany their partners to health facilities for ANC services. Similarly, facility deliveries also increased because of increased sensitisation and home visits. This is affirmed by one health care worker as follows:

*Community members have embraced this programme and adopted the interventions. Since these intervention started, we have seen women increasingly coming to the health facility and inquiring about different family planning options. They even come along with their partners. Our records also show that deliveries at this facility have increased* (IDI with health worker in MDC).

Additionally, the fact that community members participated during the first stage of implementing the interventions by selecting WGs and supervisors increased the sense of ownership of the programme. This is because they had confidence in the implementers of the interventions because they participated in selecting them. As a result, health education messages on the importance of utilisation of ANC services disseminated by the WGs were easily accepted by the community.

### The role of community leaders

Members of WGs frequently cited the enabling support of community leaders in the implementation of the interventions. Community leaders in this sense included elected and appointed leaders who formed village management teams, namely village executive officers, village chairpersons and hamlet chairpersons. These leaders were actively involved throughout the implementation phases of the interventions, and played different roles that enhanced adoption of the intervention by community members. Findings revealed that the roles played by community leaders included introducing WGs to community members during sensitisation meetings and organising meetings in their areas of jurisdiction. In these meetings, WG members delivered ANC messages. The community leaders also provided moral and material support to facilitate the work of WG members. The support was in terms of space for holding meetings and items like paper and pens. Other roles that were played by community leaders were prioritising ANC issues and integrating them in village development plans and recognising and appreciating the activities implemented by WGs. The leaders also provided security to WGs especially when visiting pregnant women at night. The same leaders were similarly instrumental to introducing the implementers of the interventions to the neighbouring village leaders when they wished to extend the implementation of the interventions. Some respondents narrated thus:

*Our village leaders accorded us all the assistance we needed. Whenever we went to their offices or called them on phone, they were always ready to help us. Our local leaders especially VEOs would even provide us with security in case we wanted to check on pregnant women at night* (IDI with MCs, in MDC).

A member of the IRT added:

*Community leaders were important during the implementation of the interventions. Given the fact that we involved them from the beginning of the interventions; it was very easy for them to support the implementation of the interventions* (IDI with IRT).

### Stakeholders’ engagement

Effective participation of stakeholders was mentioned as an important aspect that facilitated the adoption of the interventions. It was revealed that stakeholders were fully engaged through a series of workshops and review meetings held during the implementation of the interventions. In most of the meetings and workshops, the participants included district health personnel, ward and village leaders, elders, religious and traditional leaders and health care workers. During the workshops, stakeholders and WG members shared various problems, proposed interventions and reported success stories as well as revealed the challenges they encountered in their respective communities. Stakeholders also got the opportunity to give their opinions on the strategies and implementation processes. In addition, workshops served as the forum for health care workers to engage the community members and jointly refine the proposed strategies to improve ANC services in health facilities. One respondent further illustrated thus;

*Our participation during the implementation of the interventions was commendable. It enabled the understanding of what WGs were doing in the community. After being convinced by their activities as in charge of the facility, I used to ask my fellow staff to join them during sensitisation meetings so as to elaborate some issues that needed professional know-how* (IDI with health worker, in MDC).

Having witnessed the roles played by WGs through sensitization meetings and workshops, community and religious leaders joined the implementers to educate the community members in prayer houses as reported by one respondent:

*The IMCHA project was a blessing to our village. I used to participate in several review meetings where we were informed about the good work that WGs were doing in our communities. Thus, during church congregations, I would frequently preach on the significance of ANC services for our mothers and children* (IDI with CG, in MDC).

The views from the participants show that the engagement of stakeholders was crucial during the implementation of WGs interventions. In particular, it enabled the community members to know the implemented interventions in their areas of jurisdiction. Apart from providing technical knowledge during sensitization meetings, stakeholders also served as the mouthpiece for sensitising community members on the need to utilise ANC services. The engagement of community stakeholders was acknowledged by the IRT, who mentioned that this was very pivotal for establishing supporting mechanisms that encouraged, advised and helped WGs during the interventions.

### Support of local health systems

It was revealed from the findings that the success of the WGs interventions relied upon the support of local health systems. Participants reported that health workers were actively involved in different stages during the implementation of the interventions. Effective participation of health workers in workshops enabled them to not only understand the strategies implemented by the WGs to improve ANC services but also to become part of the implementation process. This was noted by one respondent;

*We worked closely with health workers in facilities and this made our work persuasive. For instance, whenever we referred pregnant women to the facility for more information, health workers would attend to them very well. Similarly, whenever we invited them to attend our community sensitisation meetings, they would come and help us in clarifying some health issues (*IDI with WGS in KDC).

This revelation was affirmed by health workers who concluded that it would be difficult for WGs to accomplish their mission without their participation:

*We are the one who received clients who were sensitised by the WGs. And in most cases, we used to attend the sensitisation meetings held at the village level; and clarified several technical ANC issues such as family planning myths* (IDI with WGS in KDC).

Community health workers, who were also Women Group Supervisors (WGSs) in this programme, were actively involved in the implementation of the interventions. Their roles were, among others, to supervise all the activities implemented by WGs, and to liaise with health workers and village leaders to ensure that all envisioned activities were implemented. They also organised sensitisation meetings, strategised the process of visiting pregnant women in households and arranged with religious leaders to visit prayer houses. WG members also received support from Health Facility Governing Committee (HFGC) members who also participated in training workshops and meetings. Apart from participating in sensitising community members on the importance of ANC, community health workers served as a bridge between the community, implementers of the interventions and health facilities.

## Factors that constrained the implementation of WGs interventions

### Physical environment

Participants complained that the implementation of the interventions was hindered by unfavourable weather conditions, coupled with topographical features of villages, as well as long distance from communities to health facilities. For instance, it was explained, that heavy rains that extended from December to May halted the implementation of most of the strategised activities. The rains caused several sensitisation meetings to be postponed and when held, attracted few community members and implementers of the interventions. One of the participants illustrated thus;

*Rains were a hindrance to our efforts in sensitising the community on the use of ANC services. We used to hold public meetings in open spaces and so, when it rained, we had to postpone the meetings. Attendance by community members in these meetings was also poor during the peak of the rainy season. On several occasions, we postponed the meetings due to rains when we had already begun. In some months, it rained consecutively for the whole week and thus disrupted our work plan* (FGD with WGS, in MDC*).*

This view was supported by some village leaders as exemplified by one respondent:

*….In our villages, especially from January to April, it rains consecutively. During this period, adequate attendance in public meetings is always challenging* (IDI with Village leader in MDC).

In addition, long distances and steep topography discouraged community members from attending sensitisation meetings. It was learnt that hamlets in which meetings were conducted were sparsely distributed such that it required reliable means of transport. The challenge of transport was said to affect the implementation of interventions since not all implementers had transport fare to reach particular hamlets. In the same vein, the steep topography in  some intervention villages affected the implementers of interventions who visited pregnant women in their households. Participants lamented that it was difficult to navigate through these communities; and this became worse when it rained as the narrow and wet pathways would be slippery.

### Mistreatment by community members

Findings in this sub-theme revealed that in some households, WGs were not jovially received; instead, they were rebuked such that some of them were about to despair carrying on the interventions. Indeed, some pregnant women and their partners thought that the WGs wanted to scout their private life. The situation worsened when WGs visited some households at night to see pregnant women who had not attended ANC. In order to reduce risks, WGs asked village leaders to offer them security while executing such tasks during the sundown. Some WG members reported that, in some households, they were even intimidated, especially during the early stage of implementing the interventions since village members had not yet clearly understood the roles of WGs in their villages. The embarrassing statements raised in the FGDs with WG members are summarized in Table 3.

**Table 3:** Embarassing statements during home visits

Kilolo district	Mufindi district
How did you come to know that I am pregnant?	You better leave my home, I have nothing to tell you
I will deal with whoever told you that I am pregnant	I don’t know why you ask me such private questions
If anything bad happens to me, you will be liable for it	Make your story very short; I have other businesses to attend to. After all, what you are saying is not new to me
My daughter is not pregnant unless you have come for other issues	My husband is not here, I cannot listen to what you are talking about. You should come next time when he is present
Give us the money that you are given instead of tormenting us with your questions	I am old enough to know my responsibility when pregnant; you are still too young to advise me

Source: Field Data (2019)

### Family-related challenges among the implementers

Families of the implementers of the interventions were mentioned as constraining the performance of the interventions as well. Firstly, not all families approved of their family members’ active participation in the implementation of the interventions. For instance, married WG members faced challenges in convincing their partners to continue implementing the interventions. Findings revealed diverse intriguing issues that surfaced among family members who disapproved of their partners’ participation in the interventions. Respondents reported that women in Kilolo and Mufindi districts are the ones who fully engage in productive activities like cultivation, thus spending more time implementing interventions could affect family food security. A participant in FGDs had this to say:

*…At times, I could not tell my husband where I was going. This is because he was not supportive of the interventions we were implementing. I knew the risk of telling him where I was going daily since I knew his reaction”* (IDI with WG, in MDC).

Secondly, some members complained about lack of compensation for the efforts made by WGs. Findings revealed that when women were selected to join WGs, their family members relaxed hoping that it was a kind of income-generating activity, only to find out that their partners were merely volunteering. According to the participants, their partners were not happy when they learnt that there would not be any payments for the tasks they were performing. One respondent elaborated thus:

*My partner always wanted to know the amount of money I was paid. No matter how often I told him that we were not paid, he never understood. You know, when you spend such a long time walking all over the village and come back home very tired with nothing in the pocket, very few partners will understand why you are still committed to the intervention* (IDI with WG, in MDC).

Thirdly, some male partners complained that WGs were not taking care of their families as they spent more time implementing the interventions. Some WG members used to meet twice a week to visit households. In most cases, sensitisation meetings were conducted in the evenings and thus WG members who lived far from the hamlets where meetings were conducted arrived home very late. This was narrated by one respondent:

*I often arrived home late because I had to walk a long distance from my home to the meeting venue. I would get back home late after the meetings and I often found my little children asleep. This most often annoyed my husband* (IDI with WG, in MDC).

Lastly, some participants revealed that family members were worried that WGs were draining family resources for the interventions. For example, in some cases, WGs had to incur transport costs when attending sensitisation meetings, and also bought stationery and drinking water during the meetings. Moreover, some more funds were needed to buy uniforms like *Vitenge (local print fabric)* and help pregnant women who needed assistance. One of the respondents explained that:

*It is true that at first when we were selected to join WGs, our partners were contented that we would bring food home; in the contrary, we took food away. This is what contributed to discontentment in our families and they did not want to hear anything regarding the interventions. At times, we did not even tell our household members where we were going to avoid escalating the conflicts* (FGD with WG, in KDC).

## Discussion of the findings

This study examined the influence of community-related factors on the performance of community-based interventions in a bid to improve ANC services. The study specifically examined how community-related factors facilitated or constrained the implementation of the interventions. It has been argued that strengthening ANC services has the potential of attaining the sustainable development goal (SDG) No. 3. The goal emphasizes that countries should have maternal mortality ratio (MMR) less than 70 per 100,000 live births by 2030 [2]. In this regard, the implementation of CBIs through WGs envisages reducing the appalling maternal deaths, especially in resource-constrained settings. The intervention strategies commonly implemented in all intervention villages included community sensitisation meetings, household visitations and sensitisation of community members in informal gatherings such as local bars and playgrounds. The strategies also subsumed formal gatherings including religious gatherings. The findings revealed that the facilitating factors included community readiness to adopt the interventions, the role of community leaders, stakeholders’ engagement and support of local health systems. On the other hand, the key factors that constrained the implementation of the community-based interventions included unfavourable physical environment, victimisation of WGs by community members and family-related challenges.

It is evident from the findings that without community readiness to adopt interventions, the implementation of the designed strategies would fail. Evidence elsewhere indicates that unless a community is ready, initiation of the interventions programme is unlikely, and if a programme starts without the support of the community, it is likely to fail [39]. Thus, tailoring community needs to intervention strategies is essential for programme success [40]. A review of 61 studies revealed that community readiness to adopt interventions becomes effective when the designed strategies do not only engage women of reproductive age and their families, but also the whole spectrum of the community for sustainable outcomes [33]. A qualitative study that employed 29 studies across 17 countries revealed that community readiness played an important role in the utilisation of maternal waiting homes to reduce maternal deaths [41]. Specifically, community readiness helped in identifying and addressing the factors limiting the use of maternal waiting homes such as getting approval and support from partners, and risks of staying away from their families for a long time [42].

The findings confirmed that effective implementation of WGs interventions to improve community health relies more on the extent to which community leaders are engaged throughout the intervention phases. The interventions implemented in Kilolo and Mufindi districts also benefited from exerted endeavours by the community leaders. A handful of studies have shown that engagement of local leaders serves as a bridge between the community, implementers of the intervention, health facilities and higher administrative structures [23, 42, 43]. In the ACCLAIM project implemented in Uganda, Malawi and Swaziland to improve maternal health, local leaders mobilised community members to seek MCH services, interacted with facility workers to ensure proper service delivery, engaged in resource mobilisation and communicated facility needs to responsible authorities [41]. It is argued that community leaders have identities of ‘trust’. For instance, informal leaders like pastors, church leaders and elders are normally trusted, while formal leaders have authority over the community; and this enables them to enforce some directives on the community [41]. In India, it was attested that when local health personnel and chairmen participated in community meetings to plan strategies with community members, discussions were livelier and planning more productive [16]. In addition, a study in Nepal reported that community leaders had direct interactions with government officials on the problems that faced health care providers and women alike; and thus, there was more openness among officials in resolving issues, and a greater willingness to accept feedback from the community [44, 45]. It has been reported that if the roles of leaders are not taken seriously, the outcomes can be compromised. This was evidenced in the intervention implemented in Uganda where local leaders failed to exert the anticipated cooperation; and thus, the performance of the intervention was slowed down [46].

The present study further showed that health facility workers, community health workers and local health facility committee members played a great role in ensuring that sensitised community members utilised the existing ANC services. Although WGs interventions are highly needed in settings without well-functioning health systems, when it happens that interventions are implemented in settings where health systems are well established, the chances that interventions will perform better are higher. In other contexts, evidence has shown that in settings where WGs interventions were implemented in communities with weak health systems, the expected outcomes were compromised. For instance, in the interventions implemented in Zambia, women volunteers expressed their dissatisfaction as many sensitized community members did not go to health facilities due to long distances [47]. Again, in Ghana, it was found out that despite the increased demand for ANC at the community level, health system challenges like delays in service provision, staff absenteeism and poor interpersonal skills deterred clients from seeking further ANC services [48]. Thus, CBIs may not yield good results if health system structures are not well mainstreamed. The findings of this study and evidence from the literature suggest that interventions aiming at improving maternal health outcomes need to be multi-pronged and comprehensive so that they explicitly address both health service constraints on the supply side, as well as contextual factors like socioeconomic factors, demographic factors and knowledge barriers, on the demand side [49, 50].

While some community factors play a pivotal role in facilitating the implementation of WGs interventions, other factors may impede the interventions. For instance, unfavourable geographical settings in which interventions are implemented have received considerable attention from scholars [30, 32, 47, 51]. In the present study, access to community sensitisation meetings and mobility within the community were a big challenge during adverse weather seasons. A study conducted in Northern Ghana reported similar findings as women volunteers and their supervisors who were supposed to visit 200 compounds faced great challenges especially during rainy seasons. They found it difficult securing transport to regularly access homes as walking was very tiring due to the long distances [48]. A study in Kenya revealed the same findings in which volunteers cited that though they were living within the community, they covered long distances on foot to reach some clients. The situation became worse when they encountered impassable roads even with the use of motorbikes [47]. In Zambia, it was reported that women group volunteers feared walking to reach distant households for referral assistance.

In order to address geographical factors like transport challenges, some interventions designed emergency means of transport ranging from motorised to non-motorised transport such as bicycles, animal-drawn carts and canoes [52–54]. Such types of  transport  played an important role in mobilising pregnant women to attend ANC thereby increasing facility delivery in Nigeria [55].

The influence of some family members like husbands or mothers-in-law in the intervention villages is also important in the utilisation of interventions. In particular, families which were not pro-interventions discouraged WGs from active involvement in the interventions thereby impeding its implementation. Families especially husbands and in-laws impede not only strategies to improve ANC services, but also the whole continuum of care to pregnant women [56, 57]. The tendency of husbands to discourage WGs members’ participation in improving ANC has been attributed to a number of issues. They include the existing gender inequality, lack of awareness due to inadequate sensitisation and limited child health education programmes in communities [57]. The findings in the present study resonate with other studies conducted elsewhere which revealed that despite women volunteers’ preference to implement the interventions; they were denied permission by their husbands and/or family members [15, 58]. The same experience of women volunteers lacking the support of their family members to implement interventions was also highlighted by Morrison et al. [15] in rural Nepal.

A number of strengths and weaknesses can be drawn from this study. The findings inform researchers, policymakers and other stakeholders about the importance of considering contextual aspects and, more importantly, community-related factors during planning, development and implementation of community-based interventions  through WGs interventions. The study also informs the importance of prioritizing community prospects, worries and opportunities when designing interventions. This study involved multiple participants; and this enabled effective validation of data from diverse sources. Besides, the qualitative design that was employed successfully captured participants’ experiences during the implementation of the WGs interventions. However, the study was not without limitations; the sampled participants were only those who actively participated during the interventions, while WGs who dropped from implementing the interventions were not included in the study. However, given the diversity among the participants and data collection techniques, exclusion of some WGs did not affect the findings because triangulation of data was enhanced. Furthermore, the limitation of this study to the two districts in Iringa Region may not reflect the prevailing community-related factors in other districts in Tanzania. Notwithstanding the limitations, this study has managed to shed light on the importance of prioritising community-related factors in designing and implementing community-based interventions  to increase adoption and sustainability of interventions.

## Conclusion

This study aimed to examine the influence of community-related factors on the performance of community-based interventions to improve ANC services. The study re-affirms that contexts in which interventions are implemented can facilitate or constrain the implementation of community-based interventions. It is evidenced that the implementation of CBIs through participatory women groups was greatly facilitated by the readiness of community members to adopt the interventions, the role of community readers, stakeholders’ engagement and support from local health systems. It was further evidenced that physical environment, mistreatment of community members and family related issues hindered smooth implementation of the interventions. As such, it is realised that the context serves as an important attribute when implementing interventions to improve ANC services. This is to say, when designing, developing and implementing community-based interventions, it is important to consider community preferences because community members are the final consumers of interventions. In other words, in-depth understanding of the community-related factors before implementation of interventions increases facilitating factors while lessening inhibiting factors during planning, development and implementation of interventions. Therefore, when designing interventions, the focus should be not only on outcomes but also on the entire implementation phases and stakeholders especially the community spectrum in which the interventions will be implemented. This may warrant adoption and sustainability of interventions.

## Data Availability

The datasets generated and/or analysed during the current study are not publicly available because they are still in use but they are available from the corresponding author on reasonable request.

## Abbreviations

ANC: Antenatal care
CBIs: Community based interventions
CCHP: Comprehensive council health plan
CG: Community Gatekeeper
CHMT: Council health management team
CHW: Community health workers
FGDs: Focus group discussion
HCP: Health care provider
HFGC: Health facility governing committee
IDI: In-depth interview
IMCHA: Innovating for maternal and child health in Africa
IRT: Implementation research team
LMIC: Low and middle-income countries
M&E: Monitoring and evaluation
MCH: Maternal and child health
PAR: Participatory action research
PLAWG: Participatory learning and action women groups
SDGs: Sustainable development goals
SSA: Sub Saharan Africa
VEO: Village executive officer
VMT: Village management team
WGS: Women group supervisors
WGs: Women groups

## References

[1] Tuncalp Ӧ, Pena-Rosas JP, Lawrie T (2017). WHO recommendations on antenatal care for a positive pregnancy experience going beyond survival. Int J Obstetrics Gynaecol.

[2] Kalipeni E, Iwelunmor J, Toussaint DG (2017). Maternal and child health in Africa for sustainable development goals beyond 2015. Glob Public Health.

[3] TTDH-MIS. Tanzania Demographic and Health Survey and Malaria Indicator Survey. Ministry of Health, Community Development, Gender, Elderly and Children (Tanzania Mainland), Ministry of Health (Zanzibar), National Bureau of Statistics, Office of the Chief Government Statistician, and ICF. Dar es Salaam, Tanzania and Rockville, Maryland. 2009.

[4] Musyoka FM, Thiga MM, Muketha GMA (2019). 24-hour ambulatory blood pressure monitoring system for preeclampsia management in antenatal care. Inf Med Unlocked.

[5] Rockers PC, Wilson ML, Mbaruku G, Kruk ME (2009). Source of antenatal care influences facility delivery in rural Tanzania: a population-based study. Maternal Child Health J.

[6] Cumber SN, Diale DC, Stanly EM, Monju N (2016). Importance of antenatal care services to pregnant women at the Buea regional hospital Cameroon. J Family Med Health Care.

[7] World Health Organization (WHO). WHO recommendation on antenatal care for positive pregnancy experience. WHO. Switzerland: Geneva. 2016. 28079998

[8] Gong E, Dula J, Alberto C, Albuquerque A, Steenland M, Fernades Q, Cuco RM (2019). Client experiences with antenatal care waiting times in southern Mozambique. BMC Health Serv Res.

[9] Shoo RS, Mboera, LEG, Ndeki S, Munishi, G. Stagnating maternal mortality in Tanzania: what went wrong and what can be done. Tanzania J Health Res. 2017;19(2):1–12.

[10] Central Statistical Agency. Ethiopia mini demographic and health survey 2014. Central Statistical Agency. Ethiopia: Addis Ababa. 2014.

[11] Uganda Bureau of Statistics (UBOS) and ICF International Inc. Uganda Demographic and Health Survey 2011. UBOS and Calverton, Maryland: ICF International Inc. Uganda: Kampala; 2012.

[12] Guttmacher R, Kelly PA, Ruiz J. Community health interventions: principles and application. John Wiley & Sons Inc. United States: San Francisco, CA. 2010.

[13] Neagi SB, Sharma J, Khanna R, Srivastava PK, Khera A, Kumar R, Zodpey S (2016). Care of newborn in the community and at home. J Perinatol.

[14] Preston R, Rannard S, Felton-Busch C, Larkins S, Camito K, Carlisle K (2019). How and why do participatory women’s groups (PWGs) improve the quality of maternal and child health (MCH) care? A systematic review protocol. BMJ Open.

[15] Morrison J, Tamang S, Mesko N, Osrin D, Shrestha B, Manandhar M (2005). Women's health groups to improve perinatal care in rural Nepal. BMC Pregnancy Childbirth.

[16] Farnworth KS, Bose K, Fajobi O, Souza PP, Peniston A, Davidson L (2014). Community engagement to enhance child survival and early development in low- and middle-income countries: an evidence review. J Health Commun.

[17] Manandhar DS, Osrin D, Shrestha BP, Mesko N, Morrison J (2004). Effect of a participatory intervention with women’s groups on birth outcomes in Nepal: a cluster-randomized controlled trial. Lancet.

[18] Colborn T, Nambiar B, Bondo A, Makwenda C, Tsetekani E (2013). Effects of quality improvement in health facilities and community mobilisation through women’s groups on maternal, neonatal and perinatal mortality in three districts of Malawi: MaiKhanda, a cluster randomised controlled effectiveness trial. Int Health.

[19] Tripathy P, Nair N, Sinha R, Rath S (2016). Effects of participatory women’s groups facilitated by accredited social health activist on birth outcome in rural eastern India: a cluster randomized controlled trial. Lancet Global Health.

[20] Azad K, Barnett S, Banerjee B, Shaha S, Khan K, Rego AR, Costello A (2010). Effect of scaling up women’s groups on birth outcomes in three rural districts in Bangladesh: a cluster-randomised controlled trial. Lancet.

[21] Ziemann A, Brown L, Sadler E, Ocloo J, Boazi A, Sandall J (2019). Influence of external contextual factors on the implementation of health and social care interventions into practice within or across countries—a protocol for a ‘best fit’ framework synthesis. Syst Rev.

[22] George AS, Branchini C (2017). Principles and processes behind promoting awareness of rights for quality maternal care services: a synthesis of stakeholder experiences and implementation factors. BMC Pregnancy Childbirth.

[23] Sondaal A, Tumbahangphe KM, Neupane R, Manandhar DS, Costello A, Morrison AC (2018). Sustainability of community-based women’s groups: reflections from a participatory intervention for newborn and maternal health in Nepal. Community Dev J.

[24] Glenton C, Colvin CJ, Carlsen B, Swartz A, Lewin S, Noyes J. Barriers and facilitators to the implementation of lay health worker programmes to improve access to maternal and child health: qualitative evidence synthesis (Review). Cochrane Database Syst Rev. 2013;10(CD010414):1–66. 10.1002/14651858.CD010414.pub2 PMC6396344 24101553

[25] McCormack B, Kitson A, Harvey G, Rycroft J, Titchen A, Seers K (2002). Getting evidence into practice: the meaning of ‘context’. J Adv Nursing.

[26] Duong DM, Bergstrom A, Wallin L, Bui H, Erikson L, Eldh AC (2015). Exploring the influence of context in community based facilitation intervention focusing on neonatal health and survival in Vietnam: a qualitative study. BMC Public Health.

[27] Pfdenhauer LM, Gerhardus A, Mozygemba K, Lysdahl KB, Booth A, Hofmann B, Rehfuess E (2017). Making sense of complexity in context and implementation: the Context and Implementation of Complex Interventions (CICI) framework. Implement Sci.

[28] Ploeg J, Wong S, Hassani K, Yous M, Fortin M, Kendall C, Liddy C, Reid MM (2019). Contextual factors influencing the implementation of innovations in community-based primary health care: the experience of 12 Canadian research teams. Primary Health Care Res Dev.

[29] Kegler MC, Rigler J, Honeycutt S (2011). The role of community context in planning and implementing community-based health promotion projects. Eval Program Plann.

[30] Kok MC, Dieleman M, Taegtmeyer M, Broerse JEW, Kane SS, Ormel H (2015). Which intervention design factors influence performance of community health workers in low- and middle-income countries? A systematic review. Health Policy Plan.

[31] Eliason R (1999). Towards sustainability in village health care in rural Cameroon. Health Promot Int.

[32] Jobson G, Naidoo N, Matlakala N, Morincowitz G, Railton J, McIntyre JA, Struthers HE, Peter RPH (2020). Contextual factors affecting the integration of community health workers into the health system in Limpopo Province, South Africa. Int Health.

[33] Miltenburg AS, van Pelt S, de Bruin W, Shields-Zeeman L (2019). Mobilizing community action to improve maternal health in a rural district in Tanzania: lessons learned from two years of community group activities. Glob Health Action.

[34] Mushi D, MpembeniRJahn A (2010). Effectiveness of community-based safe motherhood promoters in improving the utilization of obstetric care. The case of Mtwara Rural District in Tanzania. BMC Pregnancy Childbirth.

[35] Yin RK (2009). Case study research: Design and methods.

[36] Gall MD, Gall JP, Borg WR. Educational research: an introduction (8th edn). United States: New York. 2007.

[37] Maluka S, Japhet P, Fitzgerald S (2020). Leaving no one behind: using action research to promote male involvement in maternal and child health in Iringa region, Tanzania. BMJ Open.

[38] Braun V, Clarke V (2006). Using thematic analysis in psychology. Qual Res Psychol.

[39] Smith HJ, Portela AG, Marston C (2017). Improving implementation of health promotion interventions for maternal and newborn health. BMC Pregnancy Childbirth.

[40] Edward RW, Thurman PJ, Plested BA, Oetting ER, Swanson L. Community readiness: research to practice. J Commun Psychol. 2000; 28(3):291–307.

[41] Glaser E. Advancing community-level action for improving maternal, neonatal, and child health/prevention of mother-to-child HIV transmission (ACCLAIM): end of project report. Elizabeth Glaser Pediatric AIDS Foundation and ACCLAIM. Canada: Ottawa. 2017.

[42] Bjorkman M, Svendsson J (2009). Power to the people: evidence from a randomized field experiment on community-based monitoring in Uganda. Q J Econ.

[43] Ahluwalia IB, Robinson D, Valleye L, Gieseker KE, Kabakama A (2010). Sustainability of community-capacity to promote safer motherhood in northwestern Tanzania: what remains?. Glob Health Promot.

[44] Howard-Grabman L, Miltenburg AS, Marston C, Portela A (2017). Factors affecting effective community participation in maternal and newborn health programme planning, implementation and quality of care interventions. BMC Pregnancy Childbirth.

[45] Sinha D. Empowering communities to make pregnancy safer: an intervention in rural Andhra Pradesh. Health and Population Innovation Fellowship Programme. Working Paper No. 5. New Delhi Population Council. India: New Delhi. 2008.

[46] MacPhail C, Youth-Driven HIV (2006). Prevention programmes in South Africa: social capital empowerment and conscientisation. Social Dyn.

[47] Mannah MT, Warren C, Kuria S, Adegoke A (2014). Opportunities and challenges in implementing community-based skilled birth attendance strategy in Kenya. BMC Pregnancy Childbirth.

[48] Dil Y, Strachan D, Cairncross S, Korkor AS, Hill Z (2012). Motivations and challenges of community-based surveillance volunteers in the northern region of Ghana. J Community Health.

[49] Rahman S, Choudhury AA, Khanam R, Moin SMI, Ahmed S, Begum N (2017). Effect of a package of integrated demand- and supply-side interventions on facility delivery rates in rural Bangladesh: implications for large-scale programs. PLoS ONE.

[50] Dopson S, Fitzgerald LA, Dopson S, Fitzgerald LA (2005). The active role of context. Knowledge to action? Evidence-based health care in context.

[51] Jacobs C, Michelo C, Moshabela M (2018). Implementation of community-based interventions in the most rural and remote districts of Zambia: a process evaluation of safe motherhood action groups. Implement Sci.

[52] Lungu K, Kamfosa V, Hussein J, Ashwood-Smith H (2001). Are bicycle ambulances and community transport plans effective in strengthening obstetric referral systems in Southern Malawi?. Malawi Med J.

[53] Bhutta ZA, Ahmed T, Black RE, Cousens S, Dewey K, Giugliani E, Shekar M (2008). What works? Interventions for maternal and child undernutrition and survival. The Lancet.

[54] Hossain J, Ross SR (2006). The effect of addressing demand for as well as supply of emergency obstetric care in Dinajpur, Bangladesh. Int J Gynaecol Obstet.

[55] Elmusharaf K, Byrne E, O’Donovan D (2015). Strategies to increase demand for maternal health service in resource-limited settings: challenges to be addressed. BMC Public Health.

[56] Peneza AK, Maluka SO (2018). ‘Unless you come with your partner you will be sent back home’: strategies used to promote male involvement in antenatal care in southern Tanzania. Glob Health Action.

[57] Muheirwe F, Nuhu S (2019). Men’s participation in maternal and child health care in Western Uganda: perspectives from the community. BMC Public Health.

[58] Singla D, Lazarus A, Atif N, Sikander S, Bhatia U, Ahmad I, Nisar A, Khan S, Fuhr D, Pate V (2014). “Someone like us”: delivering maternal mental health through peers in two South Asian contexts. J Affective Disord.

